# Parental educational expectations and academic achievement among Tibetans in China: a chain mediation model

**DOI:** 10.3389/fpsyg.2026.1673158

**Published:** 2026-02-19

**Authors:** Licheng Shi, Yi He, Yongjiang Shen

**Affiliations:** 1 Department of Psychology, Nantong University, Nantong, China; 2 Liang Ping Middle School, Chongqing, China

**Keywords:** academic achievement, adolescents, chain mediation model, educational expectation, learning engagement

## Abstract

**Background:**

Educational expectation is a key determinant of adolescents’ academic achievement, influencing their sustained engagement in school activities. However, existing research has been controversial regarding the relationship between parents’ educational expectations and academic performance across diverse racial and ethnic groups.

**Methods:**

Based on a survey of 521 Tibetan adolescents in western China, this study examined the direct and indirect effects of perceived parental educational expectations on academic achievement. Particular attention was paid to the mediating roles of adolescents’ own educational expectations and their learning engagement. Multiple mediation analyses were conducted to test these relationships.

**Results:**

Perceptions of parental educational expectations significantly predicted their academic achievement among Tibetan adolescents. Moreover, adolescents’ educational expectations mediated the relationship between perceived parental expectations and academic achievement. Learning engagement alone did not mediate this direct relationship, but both variables exerted a chain mediation effect. Perceived parental educational expectations influenced academic achievement sequentially through Tibetan adolescents’ own educational expectations and learning engagement.

**Conclusion:**

This study confirms the chain mediation model and highlights the critical roles of both parental influence and individual psychological factors in shaping academic achievement. They have significant implications for improving the academic success of minority students such as Tibetan adolescents.

## Introduction

Educational expectations have long been recognized as a central psychological and sociocultural force shaping children’s academic development across global contexts (Li et al., 2024; Zhang et al., 2020). It refers to realistic anticipation held by parents, teachers, or adolescents themselves regarding future academic achievement, such as the highest level of educational attainment (Yamamoto and Holloway, 2010).

Globally, researched highlight parental educational expectations as an important predictor of adolescents’ academic achievement (Briley et al., 2014; Aceves et al., 2020). However, its magnitude and underlying mechanisms of this association differ a lot based on ethnic group, cultural orientation, and educational system (Cheng and Starks, 2002; Lawrence, 2015). For example, parental expectations are important factors driving upward social mobility for immigrants/minorities in the US but function through unique psychological pathways compared to majority groups (Civita et al., 2004; May and Witherspoon, 2019). The collectivistic cultural orientations in Southeast Asian heighten parental expectations on adolescents’ academic self-concept, yet limited educational resources in rural areas reduce this effect (Yeung and Xia, 2023). This global heterogeneity highlights the needs to test the generalizability of established motivational pathways to underrepresented ethnic minorities situated within unique sociocultural and educational ecologies, such as Tibetan adolescents in China.

A well-known proverb “Parents hope their children will become dragons” in China expresses the deep-rooted aspirations for youth’s academic success. However, this metaphor originates from Han cultural traditions and does not necessarily reflect Tibetan cultural norms. Nevertheless, as Tibetan families are increasingly embedded within China’s national education system, the prevailing achievement-oriented norms may shape educational expectations of both parental and adolescents, even if the cultural meanings and manifestations differ. Importantly, the vast majority of existing research has been conducted from Han-majority populations from urban or economically developed regions of China, while ethnic minority groups, particularly Tibetan adolescents, remain substantially underrepresented in the literature.

### Educational context of Tibetan adolescents

The Tibetan region, located in Qinghai Province in western China, is a minority area characterized by its remote geography and insufficient educational resources. To address these constraints, large-scale boarding school systems have been established to expand educational opportunities access for Tibetan youth over recent decades. As a result, Tibetan adolescents in boarding schools often experience prolonged periods of limited daily contact with their parents due to long travel distances and geographic remoteness (Tong and Zhou, 2023), which may shape how Tibetan adolescents perceive and internalize parental educational expectations (Yang et al., 2021).

Notably, Tibetan adolescents’ academic achievement, on average, lags behind that of Han peers in urban and economically developed regions (Li et al., 2024; Yang et al., 2021). Yet, research on the psychological and social factors shaping Tibetan adolescents’ academic achievement remains scarce. Existing scholarship predominantly focuses on structural barriers (e.g., resource allocation, policy interventions) rather than individual or family-level motivational mechanisms, creating a critical gap in understanding how factors like educational expectations might mitigate structural disadvantages.

### Expectancy–Value Theory (EVT)

The Expectancy–Value Theory (EVT) stands as a foundational psychological framework for understanding how psychological characteristics interact to motivation and task success (Wigfield and Eccles, 2000; Briley et al., 2014). According to the EVT, achievement outcomes are jointly influenced by adolescents’ expectancy beliefs, socialization influences (e.g., expectations particularly from parents), and prior academic experiences (Lawrence, 2015; Wu and Fan, 2017). Within this framework, parental expectations are regarded as a pivotal social contextual factor that shapes adolescents’ academic self-perceptions and aspirations (Yamamoto and Holloway, 2010). That is, when parents hold high expectations for their children’s academic success, such expectations are internalized by adolescents, fostering more positive competence beliefs and higher educational aspirations. These internalized beliefs, in turn, promote greater behavioral investment in learning (e.g., learning engagement) and ultimately better academic achievement (Zhang et al., 2011).

Thus, EVT provides a clear theoretical rationale for examining whether parental expectations influence Tibetan adolescents’ achievement through internal motivational and behavioral mediators.

Prior research has validated this pathway in Han-majority populations in China. For example, Zhang et al. (2011) found that Han adolescents’ own educational expectations mediated the relationship between parental expectations and academic achievement, with learning engagement acting as a downstream behavioral mediator. Similar findings have been reported in other East Asian contexts (Wu and Fan, 2017; Yeung and Xia, 2023) and Western minority groups (Aceves et al., 2020; May and Witherspoon, 2019). However, no studies have explicitly tested whether this expectancy-based pathway extends to Tibetan adolescents.

### Perceived parental educational expectation and academic achievement

Research across diverse racial and ethnic groups indicates that higher parental expectations are associated with better academic achievement (Zhang et al., 2011; Aceves et al., 2020). In general, parental expectations have been found to play a critical role in children’s academic success. From this perspective, adolescents’ academic trajectories and interactions with the educational system are shaped by the internalization of parental beliefs. Optimistic parental educational expectations can foster adolescents’ higher academic achievement than would be predicted based solely on a family’s socioeconomic background (Bui, 2019; Pinquart and Ebeling, 2020a).

However, some studies find significant variation across ethnic and socioeconomic groups in the effect of parental expectations on student outcomes. Parents from minority racial backgrounds and low-income families may hold higher educational expectations for their children, which is also an important way for their children to succeed in their studies and achieve upward social mobility (Civita et al., 2004). The reasons why minority parents maintain high expectations despite lower academic achievement remain unclear, though one possible explanation is that these expectations may be unrealistic. Otherwise, the correlation of parental expectations to concurrent or future student achievement outcomes tends to weaker for racial/ethnic minority families (Cheng and Starks, 2002; Yamamoto and Holloway, 2010; Lawrence, 2015).

A meta-analysis by Pinquart and Ebeling (2020a) identified a moderate association between success expectations and both current (*r* = 0.34) and future academic achievement (*r* = 0.41). Parents’ high educational expectations, while positively correlated with later outcomes, are not a sufficient condition for high educational attainment for any social group (Pinquart and Ebeling, 2020a; Urhahne and Wijnia, 2023). Other researchers have demonstrated reciprocal relationships between academic achievement and perceived parental educational expectations (Zhang et al., 2011; Bui, 2019).

For Tibetan families with geographical and resource constraints, parental educational expectations proved to be symbolic indicators of social mobility, hence maintain a prolonged motivational force even when daily interaction is limited (Yamamoto and Holloway, 2010; Briley et al., 2014). In the boarding-school contexts, these expectations act as the relatively stable reference points that guide adolescents’ educational self-concepts and aspirations, which presents an theoretical foundation to the study of expectancy-based motivational pathways. Based on prior evidence, we propose the following hypothesis: **H1: Among Tibetan adolescents, perceived parental educational expectations positively predict adolescents’ academic achievement.**

### Mediating role of adolescents’ educational expectations

Parental Educational Expectations, as a crucial factor psychological environment in family, can both be a source of academic support and a potential stressor among adolescents (Rimkute et al., 2012; Chen et al., 2022). Researches indicates that adolescents’ educational expectations were strongly connected to the educational expectations of their parents (Pinquart and Ebeling, 2020b). Longitudinal studies offer evidences that parental expectation is causal determinant of adolescent’ educational expectation and academic achievement, buffering the influence of family background, prior achievement and low teacher expectations (Benner and Mistry, 2007; Yamamoto and Holloway, 2010). This influence extends across different developmental stages, from childhood (Gill and Reynolds, 1999) to adolescent and young adulthood (Tynkkynen et al., 2012). Parents play key roles in shaping their children’s academic trajectories by instilling the value of education and giving them the confidence that they can attain success in education. Furthermore, adolescents’ perceptions of parental expectations are crucial in shaping and reinforcing their own academic self-concept and motivation (Briley et al., 2014).

However, prior research also suggests that parental educational expectations do not always function uniformly as a positive influence. As adolescents mature, expectations may increasingly be evaluated in light of their own abilities and academic realities, and in some cases may be experienced as psychological pressure rather than motivational support (Eccles and Wigfield, 2002; Chen et al., 2022). Importantly, such findings highlight that the effects of parental expectations may depend on how they are perceived and internalized by adolescents.

Some studies have demonstrated that the relationship between perceived parental educational expectations and academic achievement is statistically mediated by adolescents’ educational expectations, and this finding has been widely replicated (Simpkins et al., 2012; Briley et al., 2014; Aceves et al., 2020). However, empirical studies suggest that parental and adolescent educational expectations are not always fully aligned, as adolescents do not passively adopt their parents’ expectations. This divergence may represent a normative aspect of adolescent development (Wang and Benner, 2014). In a study on ethnic minority families, Dong et al. (2009) found that, compared to Han’s parents, the educational expectations of ethnic minority students were less influenced by parental expectations. However, little is known about how parents’ and adolescents’ educational expectations work in conjunction across different demographic groups (e.g., Zhang et al., 2011). Building on this gap, the present study proposes the following hypothesis: **H2: Among Tibetan adolescents, we hypothesize that adolescents’ educational expectations mediate the relationship between perceived parental expectations and academic achievement.**

### Mediating role of learning engagement

Learning engagement is a sustained and positive emotional state that students exhibit throughout the learning process, which is a multifaceted concept, encompassing behavioral, emotional, psychological, and cognitive components (Appleton et al., 2010). EVT suggests that educational expectations and values translate into achievement partly through motivational behaviors such as persistence, concentration, and enthusiasm. Although some researchers have shown that the more students invest in their studies, the higher their academic achievement tends to be (Cox and Marshall, 2020; Zhang et al., 2020; Çali et al., 2024). Some researchers have linked a lack of learning engagement to the emergence of bored, unmotivated, and disengaged adolescents (Appleton et al., 2010). The influence of learning engagement on academic achievement is well established. It is not only a key indicator for assessing the quality of education and student development, but also a critical determinant of academic success (Lee, 2014; Çali et al., 2024). However, little is known about its role among Tibetan students.

Most studies have confirmed that adolescents who perceive higher educational expectations from their parents tend to exhibit greater academic motivation and are more willing to participate in extracurricular activities (Beal and Crockett, 2010; Domina et al., 2011). This heightened engagement fosters a sense of competence and increases the likelihood of academic success (Wigfield and Eccles, 2000; Bouchey and Harter, 2005; Gniewosz and Noack, 2012; Wu and Fan, 2017; May and Witherspoon, 2019). Meanwhile, adolescents’ perceptions of parental educational expectations can negatively predict academic burnout while simultaneously enhancing learning engagement and academic achievement (Salmela-Aro and Upadyaya, 2017; Luo et al., 2023). In light of this, we test whether learning engagement serves as an additional mediator: **H3: Among Tibetan adolescents, we hypothesize that learning engagement plays a mediating role between perceived parental educational expectation and academic achievement.**

### Chain mediation of expectations and engagement

Expectancy–Value Theory implies a sequential pathway that parental expectations may elevate adolescents’ own expectations, which in turn stimulate higher learning engagement. Prior research has examined the interrelationships among parental educational expectations, adolescents’ own educational expectations, and learning engagement across diverse populations, including typically developing youth and children with disabilities (Yeung and Xia, 2023; Cox and Marshall, 2020). Adolescents with high academic expectations tend to invest more time in their studies, actively pursue knowledge, and work diligently to achieve their academic goals (Zhang et al., 2011; Lee, 2014; Yeung and Xia, 2023). Parental educational expectations can affect learning engagement indirectly through adolescents’ educational expectations (Cox and Marshall, 2020; Pinquart and Ebeling, 2020a; Zhang et al., 2020). However, these findings remain largely untested among Tibetan, where direct parental behavioral involvement is limited, that may alter their motivational impact.

**FIGURE 1:**
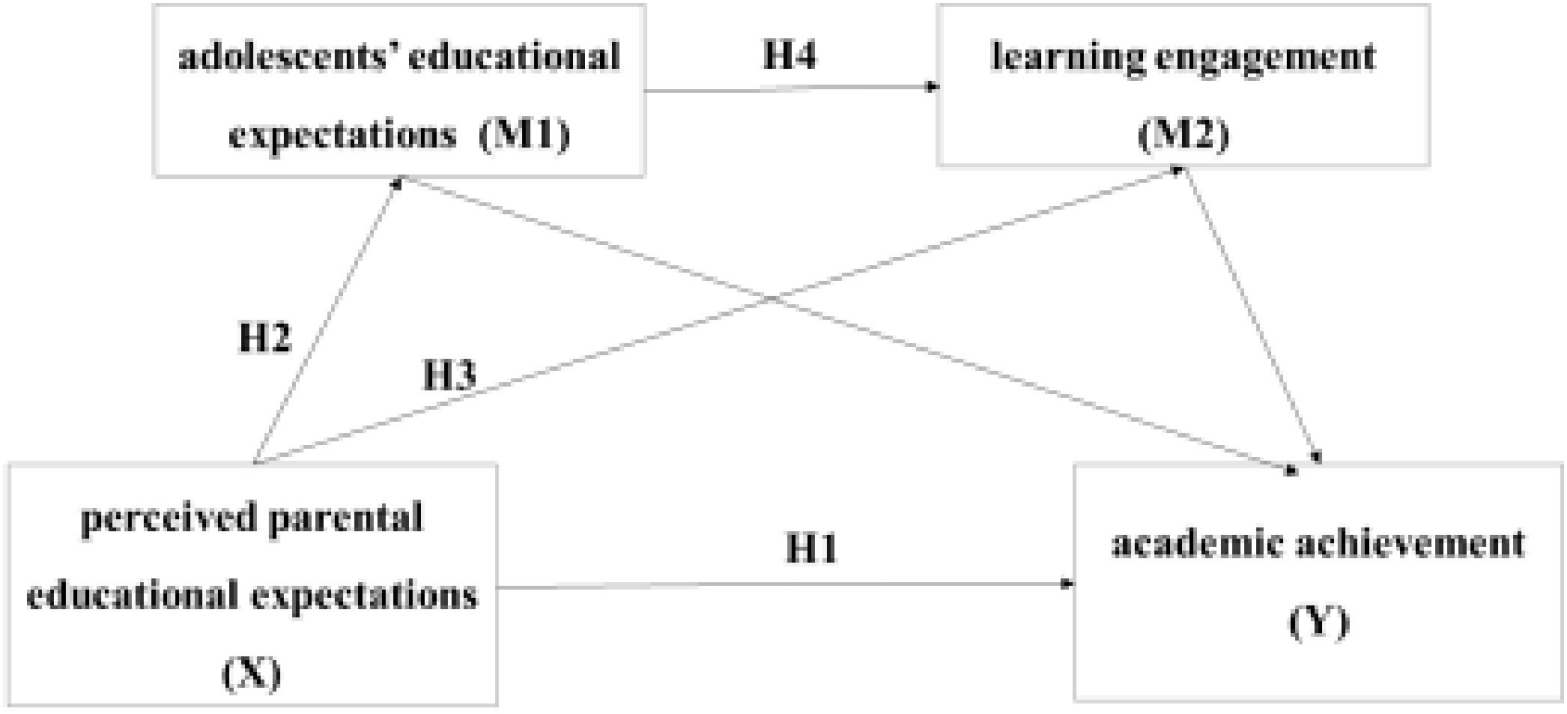
Conceptual model with hypothesized paths (H1–H4). H1: X (H1–H4)del with 1-5g parents’ educational expectations,.

Based on these studies, the present study proposes the following hypothesis: H4: Among Tibetan adolescents, we hypothesize that perceived parental educational expectations have positive impact on academic achievement through a chain mediation of adolescents’ educational expectations and learning engagement (Figure 1).

As shown in Figure 1, H1–H4 are labeled on the corresponding paths to clarify the conceptual directions of the hypothesized relationships.

Accordingly, grounded in Expectancy–Value Theory (EVT), this study aims to validate the contextual applicability of the expectancy-based motivational pathway to this historically underrepresented ethnic minority group, and advances a more precise psychological mechanism linking family-level expectations to individual academic achievement.

## Materials and methods

### Ethics statement

This study was approved by the Academic Ethics Committee of Nantong University (Approval No.: 2024-229). All procedures conformed to the Declaration of Helsinki. Given that the participants were minors (13–15 years old), written informed consent was obtained from their parents or legal guardians via school-administered consent forms. Verbal assent was also obtained from each adolescent after the research team provided a detailed explanation of the study aims, procedures, and anonymity guarantees. All questionnaires were completed anonymously. All datasets were stored on an encrypted drive, and shared only in aggregate form.

### Research design

This study adopted a cross-sectional survey design to examine the serial mediation relationships among perceived parental educational expectations, adolescents’ own educational expectations, learning engagement, and academic achievement. Data were collected between March and April 2025 in a Tibetan boarding school located in Qinghai Province, western China. The survey was administered during regular class hours, with two trained researchers present to provide standardized instructions, answer questions, and ensure participants understood the anonymity and voluntary nature of the study. Each participant completed the questionnaire independently (approximately 15–20 min per person), and all questionnaires were sealed in envelopes immediately after completion to protect privacy.

### Participants

The study participants were drawn from a Tibetan boarding school in western China, and a cluster sampling method was used at the class level. We invited all intact seventh and eighth grade classes to avoid selection bias. Notably, the selected school is one of the most representative boarding schools in the local Tibetan area.

The initial sample consisted of 549 students. We implemented strict screening criteria to ensure data quality: (1) questionnaires with missing values for key variables exceeding 10% were excluded; (2) responses with obvious response bias (e.g., uniform ratings for all items, inconsistent answers to logically related questions) were eliminated. Ultimately, 521 valid samples were retained (valid rate = 94.9%). Among them, 278 were in the seventh grade (53.36%), and 243 were in the eighth grade (46.64%), with a mean age of 14.08 years old (SD = 0.53). The sample included 236 boys, representing 45.30% of the total (See Table 1). And the study was approved by the ethics committee of the local university.

**TABLE 1:** Sample characteristics (*N* = 521).

Variable	Category/description	*n*	%
Total valid cases		521	
Missing cases	Incomplete questionnaires excluded	28	
Gender	Boys	236	45.30%
	Girls	285	54.70%
Grade level	7th grade	278	53.36%
	8th grade	243	46.64%
Age	Mean (SD)	14.08 (0.53)	

### Materials

#### Perceived parental educational expectation

Perceived parental educational expectations were assessed using the Chinese version of the Perceived Parental Expectations Scale (PPES) (Wang and Benner, 2014; Guo et al., 2017). The scale consists of 1 item that assesses adolescents’ perceptions of their parents’ educational expectations for them (“How far in school do you think your father and your mother want you to get?”). Response options ranged from 1 (less than high school), 2 (junior high school/technical secondary school), 3 (high school/vocational, trade, business school), 4 (attend college), to 5 (more than 4-years college). Higher scores indicated greater parental expectations for education.

#### Adolescents’ educational expectations

Adolescents’ educational expectations were measured using the Chinese version of the Adolescents’ Educational Expectations Scale (EES) (Wang and Benner, 2014; Guo et al., 2017). They reported their own educational expectations for themselves (“How far in school do you think you will go?”) by choosing one of the following alternatives: 1 (less than high school), 2 (junior high school), 3 (high school/vocational school), 4 (college) and 5 (more than 4-years college). The response options were consistent with PPES.

#### Learning engagement

The Chinese version of the Learning Engagement Scale (LES), revised by Fang et al. (2008), was employed to assess learning engagement. The scale comprises 17 items and evaluates three dimensions: vitality (6 items, e.g., “When I study, I feel energized”), dedication (5 items, e.g., “learning is purposeful and meaningful”), and focus (6 items, e.g., “When I study, I forget everything around me”). Each item was rated on a 7-point Likert scale, ranging from “Never” (1 point) to “Always” (7 points), with higher scores indicating greater learning engagement. Following common practice, composite scores were computed as the mean of all items, and subscale scores were calculated as the mean of items within each dimension. The scale demonstrated high reliability in this study, with a Cronbach’s alpha coefficient of 0.91 for the overall scale and 0.74–0.83 for the individual dimensions. Previous research has confirmed its effectiveness in measuring adolescents’ learning engagement.

#### Academic achievement

In this study, academic achievement was assessed using standardized test scores. We calculated an overall measure of academic achievement by averaging test scores from reading, mathematics, physics, history, geography, and biology courses (e.g., Deary et al., 2007; Grammatikopoulou, 2026). To control for grade-level effects, the original scores were converted into grade-level standard scores, following the method outlined by Cheung and Pomerantz (2015). The academic achievement composite demonstrated acceptable internal consistency in the current sample (Cronbach’s α = 0.784).

#### Data analysis

Data collection and analysis for this study were conducted using IBM SPSS Statistics version 25.0. First, descriptive statistics, including means and standard deviations, were calculated for the primary variables. Second, Pearson correlation analysis was performed to examine the bivariate relationships among the four variables, with statistical significance determined based on conventional thresholds (Cohen, 1988). Gender and grade level were included as control variables throughout the subsequent analyses to account for potential confounding effects. Finally, we used Model 6 of the PROCESS macro for SPSS (Hayes, 2013) to test the serial multiple mediation effect, and adopted bias-corrected bootstrap sampling (5,000 resamples) to estimate 95% confidence intervals for indirect effects. This method is widely recommended for mediation analysis due to its robustness to non-normal data (Preacher and Hayes, 2008).

#### Assessment of common method bias

Since key variables such as perceived parental educational expectations, adolescents’ educational expectations, and learning engagement were assessed via self-report, we then conducted Harman’s single-factor test as a preliminary diagnostic assessment for common method bias (Podsakoff et al., 2003; Zhou and Long, 2004). Results showed that the interpretation ratio of the first common factor was 31.84%, which was below the critical value of 40%. This indicated that there was no significant common method bias in this study and further data analysis could be carried out.

## Results

### Descriptive statistics and intercorrelation analysis

Means, standard deviations, and intercorrelations for each variable are shown in Table 2. The means and standard deviations (M ± SD) of perceived parental educational expectation, adolescents’ educational expectations, learning engagement, and academic achievement were 4.02 ± 1.06, 3.93 ± 1.08, 4.88 ± 0.97, 47.73 ± 15.02, respectively. Previous studies have found that there may be discrepancies between adolescents’ and parents’ views on educational expectation (Wang and Benner, 2014). In this study, adolescents perceived their parents’ expectations to be slightly higher compared to their own expectations. Most parents may want their children to go to college (*M* = 4.02, SD = 1.06), although some of adolescents expected to attain postsecondary education (*M* = 3.93, SD = 1.08). The discrepancy scores reiterated these findings that parents held slightly higher expectations than adolescents.

**TABLE 2:** Descriptive analysis and intercorrelation analysis (*N* = 521).

Variable	M ± SD	1	2	3	4
1. Perceived parental educational expectation	4.02 ± 1.06	1	1	1	
2. Adolescents’ educational expectation	3.93 ± 1.08	0.68**			
3. Learning engagement	4.88 ± 0.97	0.27**	0.35**		
4. Academic achievement	47.73 ± 15.02	0.21**	0.32**	0.31**	1

**p < 0.01.

Correlation analysis revealed that perceived parental educational expectation was significantly correlated with and adolescents’ educational expectation (*r* = 0.68, *p* < 0.01), learning engagement (*r* = 0.27, *p* < 0.01), and academic achievement (*r* = 0.21, *p* < 0.01). adolescents’ educational expectation also had significantly positive correlations with learning engagement (*r* = 0.35, *p* < 0.01) and academic achievement (*r* = 0.32, *p* < 0.01). In addition, learning engagement was positively correlated with academic achievement (*r* = 0.31, *p* < 0.01). These results suggest that further exploration of mediation effects is warranted. As displayed in Table 2.

We conducted additional non-linear sensitivity analyses to examine the robustness of the findings. Curve estimation analyses showed that the linear model fit the data well (R^2^ = 0.127, *p* < 0.001). Although the quadratic model yielded a marginally higher R^2^ (R^2^ = 0.138), the improvement was negligible, and the cubic model provided no additional benefit. These findings support the adequacy of a linear specification.

### Mediation effects analysis

The regression analysis results revealed that perceived parental educational expectations significantly and positively predicted academic achievement (β = 0.188, 95% CI [0.116, 0.277]) when no mediating variables were included. Hypothesis 1 has been supported.

After incorporating the mediating variables of adolescents’ educational expectations, the analysis revealed that perceived parental educational expectation significantly and positively predicted the Tibetan adolescents’ educational expectations (β = 0.686, *p* < 0.001). Additionally, adolescents’ educational expectations significantly and positively predicted the academic achievement (β = 0.236, *p* < 0.001). Although perceived parental educational expectation did not directly predict academic achievement, the indirect effect of perceived parental educational expectation, via adolescents’ educational expectations, on academic achievement was significant (β = 0.162, 95% CI [0.087, 0.237]). In other words, Tibetan adolescents who perceived higher levels of educational expectation from parents tended to have higher self-expectations, which in turn led to greater academic achievement. Hypothesis 2 was verified.

However, perceived parental educational expectation did not significantly predict learning engagement (β = 0.059, *p* = 0.252), nor did it indirectly predict academic achievement through learning engagement (β = 0.013, 95% CI [−0.012, 0.040]). Consequently, learning engagement did not significantly mediate the relationship between perceived parental educational expectation and academic achievement. Hypothesis 3 was not supported. Overall, the pattern of results suggests a potential case of inconsistent mediation (suppression). Although perceived parental educational expectations were positively correlated with learning engagement at the zero-order level (*r* = 0.27, *p* < 0.01), the direct path from parental expectations to learning engagement became non-significant after adolescents’ educational expectations were included. In contrast, parental expectations strongly predicted adolescents’ expectations (β = 0.686, *p* < 0.001), which in turn predicted learning engagement (β = 0.253, *p* < 0.001), indicating that the effect of parental expectations on engagement operates primarily through adolescents’ internalized expectations. Sensitivity analyses supported this interpretation. Parental expectations significantly predicted learning engagement when adolescents’ expectations were excluded (β = 0.27, *p* < 0.001) and in a parallel mediation model (β = 0.26, *p* < 0.001), but not when the serial pathway was specified. Collinearity diagnostics indicated no problematic multicollinearity (VIFs = 1.23–1.89). These findings support a theoretically meaningful suppression pattern.

Furthermore, the chain mediation analysis indicated that adolescents’ educational expectations significantly and positively predict the learning engagement (β = 0.253, *p* < 0.001). Perceived parental educational expectation indirectly affect academic achievement through the chain mediating effect of adolescents’ educational expectations and learning engagement (β = 0.040, 95% CI [0.020, 0.066]). These results demonstrate that the effect of perceived parental educational expectation on academic achievement was fully mediated through adolescents’ educational expectations and learning engagement, thereby verifying Hypothesis 4.

**FIGURE 2:**
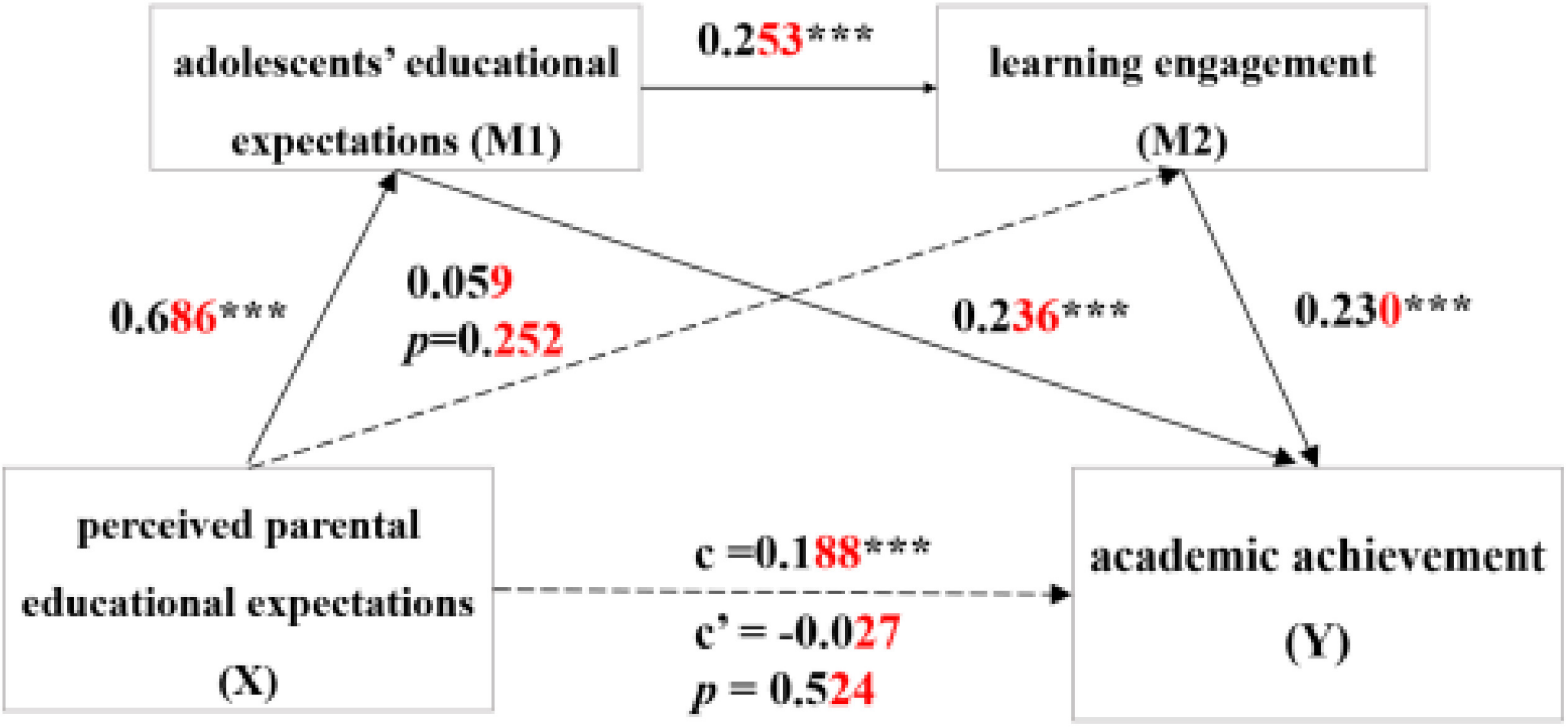
Chain mediation model between adolescents’ educational expectations and learning engagement in the relationship between perceived parental educational expectation and academic achievement. ***p < 0.001, significant regression coefficient.

Mediation analysis results are shown in Figure 2 and Table 3.

**TABLE 3:** Bootstrap analysis of mediation effects.

Path		β	Boot*SE*	95% CI
Indirect effect 1 Mediating effect 1 (hypothesis 2)	Perceived parental educational expectation → adolescents’ educational expectations → academic achievement X → M1 → Y	0.162	0.039	[0.087, 0.237]
Indirect effect 2 Mediating effect 2 (hypothesis 3)	Perceived parental educational expectation → learning engagement → academic achievement X → M2 → Y	0.013	0.013	[−0.012, 0.040]
Indirect effect 3 Chain mediating effect (hypothesis 4)	Perceived parental educational expectation → adolescents’ educational expectations → learning engagement → academic achievement X → M1 → M2 → Y	0.040	0.012	[0.020, 0.066]
Total indirect effect		0.215	0.039	[0.139, 0.294]
Direct effect		−0.027	0.052	[−0.130, 0.060]
Total effect		0.188	0.041	[0.116, 0.277]

β is the standardized regression coefficients. BootSE is the bootstrapping standard error. Empirical 95% confidence interval does not overlap with zero.

## Discussion

This study examined how perceived parental educational expectations influence academic achievement among Tibetan adolescents through multiple serial mediation chain. The findings indicated that perceived parental educational expectation was a significant predictor of adolescents’ academic achievement. Specifically, higher perceived parental educational expectation enhanced adolescents’ own educational expectations and greater learning engagement, which in turn contributed to higher academic achievement. These findings identify an expectancy-focused mechanism grounded in Expectancy–Value Theory (EVT) and support the contextual validity of EVT’s expectancy-focused pathways in a historically underrepresented group, namely Tibetan boarding-school adolescents.

### The impact of perceived parental educational expectation on academic achievement

The Tibetan adolescents in this study reported high levels of perceived parental educational expectations and self-educational aspirations, despite their parents’ low economic status and limited educational backgrounds. Furthermore, this study verified that perceived parental educational expectations could positively impact adolescents’ academic achievement, aligning with previous research across various age groups (Pinquart and Ebeling, 2020a; Smyth, 2020; Hemmerechts et al., 2022) and nationalities (Lawrence, 2015; May and Witherspoon, 2019; Aceves et al., 2020; Li et al., 2024). Importantly, the present findings reflected linear associations between perceived parental educational expectations and adolescents’ academic achievement. While prior studies have suggested that excessively high expectations may function as psychological pressure and potentially undermine motivation, such effects are likely to emerge under specific conditions in which expectations are perceived as unrealistic or controlling (Simpkins et al., 2012; Chen et al., 2022).

Despite being selected from Tibetan boarding schools and having limited daily contact with their parents, these adolescents are still influenced by their parents’ educational expectations, which is consistent with prior empirical work. This relationship is noteworthy given the unique circumstances of Tibetan boarding schools, where adolescents experience limited daily contact with their parents due to geographical barriers. Among Tibetan adolescents in boarding-school contexts, parental educational expectations may carry particular symbolic meaning as indicators of family investment and aspirations for upward mobility. When internalized in this manner, expectations appear to support rather than undermine adolescents’ academic development. This finding underscores the enduring role of parental expectations as an important motivational cue, even in remote or resource-limited minority regions (Bui, 2019; Curran and Hill, 2022).

### Mediating role of adolescents’ educational expectations

The results find that adolescents’ educational expectations fully mediate the relationship between perceived parental educational expectations and academic achievement. Specifically, parental educational expectations do not directly predict adolescents’ academic achievement. Rather, they shape adolescents’ own educational expectations, which subsequently lead to improved academic performance. This finding aligns with Expectancy–Value Theory (EVT) and previous research (Aceves et al., 2020; May and Witherspoon, 2019), which emphasizes that adolescents’ academic achievement is shaped by their expectancy beliefs and the internalization of significant others’ expectations (Wigfield and Eccles, 2000; Yamamoto and Holloway, 2010).

An important theoretical implication of the present findings lies in clarifying how parental educational expectations are internalized among Tibetan adolescents in boarding-school settings. This internalization-based mechanism helps explain why perceived parental educational expectations remain strongly associated with adolescents’ own educational expectations and academic achievement, even in contexts characterized by physical separation from parents (Yamamoto and Holloway, 2010; Briley et al., 2014). Taken together, these findings support an expectancy-focused pathway in which parental expectations influence academic achievement primarily through adolescents’ internal belief systems rather than through direct parental involvement.

### Mediating role of learning engagement

The results indicate that learning engagement does not independently mediate the relationship between perceived parental educational expectations and academic achievement among Tibetan adolescents. Hypothesis 3 (H3) was not supported. These findings are inconsistent with some previous research (Cox and Marshall, 2020). However, this does not imply that learning engagement is irrelevant in the motivational process. Instead, the results suggest a more nuanced mechanism in which parental expectations influence learning engagement primarily through adolescents’ internalized educational expectations. In other words, adolescents must internalize these expectations for them to function as a source of motivation (Kirk et al., 2011). This finding aligns with core assumptions of Expectancy–Value Theory, which emphasizes that behavioral outcomes such as effort and persistence are driven primarily by individuals’ own expectancy beliefs rather than by external influences (Wigfield and Eccles, 2000). In the present context, parental expectations appear to shape adolescents’ motivation by first shaping how adolescents see their own educational futures, which subsequently promotes greater engagement in learning activities. Thus, learning engagement functions as a downstream behavioral manifestation of internalized expectations rather than a direct response to perceived parental expectations.

However, learning engagement can significantly and positively predict academic achievement. For Tibetan adolescents, higher levels of learning engagement correspond with better academic performance, which is consistent with existing research results (Yu et al., 2022). This highlights the importance of increasing learning engagement to improve academic achievement among Tibetan adolescents.

### Chain mediating effect of adolescents’ educational expectations and learning engagement

The results indicate that Tibetan adolescents’ educational expectations and learning engagement play chain mediating roles between perceived parental educational expectation and academic achievement. As important others of adolescents, parents influence their children’s educational expectations and aspirations through their attitudes and values regarding education. Adolescents internalize their parents’ behaviors, attitudes, and values through family socialization, and then shape their own educational values. Adolescents with higher educational expectations generally exhibit greater motivation for learning, leading to higher levels of learning engagement. Furthermore, Tibetan adolescents’ educational expectations directly influence their academic achievement, and indirectly affect it through learning engagement, which corroborates the findings of Zhang et al. (2020). This sequential pathway highlights engagement as a downstream behavioral expression of adolescents’ internal motivational beliefs.

In addition to EVT, the Social Cognitive Theory (SCT; Bandura, 2001) and Self-Determination Theory (SDT; Deci and Ryan, 2000) also shed light on the mechanisms linking parental expectations to learning engagement and academic achievement. SCT also places much importance on self-efficacy as an intermediary between social factors (e.g., parental expectations) and behavioral outcomes. Academic self-efficacy in teenagers can be influenced by parental expectations as they may give implicit feedback on the potential of the adolescents (e.g., my parent believes I can succeed so I can as well). The increased self-efficacy, however, encourages the increased engagement in learning (e.g., persistence, effort) and eventually leads to the higher academic achievement. From SDT perspective, intrinsic motivation in adolescents can be instilled by psychological needs, and parental educational expectations can accomplish this. Among Tibetan adolescents in boarding schools, perceived parental expectations might act as a symbolic relatedness, which strengthens their feeling of being regarded and belonging to their families and thus increasing the autonomous learning motivation (Deci and Ryan, 2000).

In summary, the present findings indicate that parental educational expectations exert an indirect, psychologically mediated influence on Tibetan adolescents’ academic achievement. Specifically, adolescents first internalize parental expectations as their own educational aspirations, which in turn motivate heightened learning engagement and ultimately foster improved academic outcomes.

This paper verifies a motivational path under an expectance-based model for Tibetan boarding-school adolescents. The findings underscore its cross-contextual robustness. At the same time, it improves the theoretical model by showing that learning participation is not an independent mediator between parents’ expectations and children, but rather a form of downstream behavior that adolescents exhibit after internalizing their own internalized expectancy beliefs.

Beyond the theoretical contributions, the findings offer practical significances for ethnic minority and resource-constrained. First, interventions targeting Tibetan adolescents’ academic achievement should prioritize strengthening their own educational expectations over sole focus on parental involvement. Second, teachers and counselors from boarding schools can serve as a compensatory function to translate parental aspirations into students’ internal motivation, particularly given limited parent-adolescent daily contact. Finally, when policymakers create educational support programs, they should recognize that psychological factors, such as expectations and engagement, are significant levers for academic achievements. Interventions incorporating academic support with motivational development are likely to yield more sustainable, long-term benefits for minority adolescents.

### Limitations and future research

The current study explores the underlying mechanism linking perceived parental educational expectation to adolescents’ academic achievement, and provides theoretical and practical implications in Tibetan regions. Conducted in minority areas, the study enriches the literature by highlighting the connection between adolescents’ own academic expectations and perceived parental expectations, and its effect on learning engagement and academic achievement. The findings offer practical suggestions on how to improve academic achievement of Tibetan youth.

Despite these contributions, several limitations should be acknowledged. First, the participants were drawn from a single Tibetan region in western China, which may limit the generalizability of the findings to other ethnic minority populations. Future studies may further broaden the sampling scope and expand to multiple Tibetan schools to improve generalizability to enhance the robustness and external validity of the results. Second, due to administrative restrictions of boarding schools, information such as socioeconomic status and parental characteristics could not be obtained from school records, nor could parents be directly contacted. The data is collected solely through adolescents’ self-reports, which is susceptible to social desirability or subjective bias. Future research may employ multi-informant data collection (e.g., parent questionnaires or administrative records from education bureaus) to obtain these family-level variables and provide a more comprehensive understanding of the factors involved. Finally, as suggested by EVT, expectations may increasingly be experienced as pressure as children mature. Future research should examine potential non-linear associations or moderation processes, such as whether perceived parental pressure alters the strength or direction of the expectancy–achievement relationship, thereby specifying when expectations function as a value cue versus a psychological cost. At the same time, future research should explore how expectancy, value, and cost interact to influence the academic performance of Tibetan adolescents, thereby advancing the development of Expectancy-Value Theory.

## Conclusion

This study confirms that perceived parental educational expectations significantly predict academic achievement among Tibetan adolescents. Furthermore, adolescents’ own educational expectations were found to mediate the relationship between perceived parental expectations and academic achievement. Additionally, the combination of adolescents’ educational expectations and learning engagement played chain mediating roles in this relationship. These findings offer valuable insights for educators, practitioners, and policymakers seeking to promote educational expectations and learning engagement, thereby enhancing academic achievement in Tibetan adolescents.

## Data Availability

The raw data supporting the conclusions of this article will be made available by the authors, without undue reservation.
